# Ontological Problem-Solving Framework for Assigning Sensor Systems and Algorithms to High-Level Missions

**DOI:** 10.3390/s110908370

**Published:** 2011-08-29

**Authors:** Joseph Qualls, David J. Russomanno

**Affiliations:** 1 Department of Electrical and Computer Engineering, Herff College of Engineering, University of Memphis, 3720 Alumni Avenue, Memphis, TN 38152, USA; 2 Department of Electrical and Computer Engineering, Purdue School of Engineering and Technology, Indiana University-Purdue University Indianapolis (IUPUI), 799 W. Michigan St., Indianapolis, IN 46202, USA; E-Mail: drussoma@iupui.edu

**Keywords:** sensor networks, Sensor Ontology, profiling sensors, mission tasking

## Abstract

The lack of knowledge models to represent sensor systems, algorithms, and missions makes opportunistically discovering a synthesis of systems and algorithms that can satisfy high-level mission specifications impractical. A novel ontological problem-solving framework has been designed that leverages knowledge models describing sensors, algorithms, and high-level missions to facilitate automated inference of assigning systems to subtasks that may satisfy a given mission specification. To demonstrate the efficacy of the ontological problem-solving architecture, a family of persistence surveillance sensor systems and algorithms has been instantiated in a prototype environment to demonstrate the assignment of systems to subtasks of high-level missions.

## 1. Introduction

Dynamically discovering, matching, and integrating sensors and compatible algorithms to form a synthesis of systems that are capable of satisfying subtasks of high-level missions poses a significant challenge for network-centric architectures. Compounding the challenge is the lack of knowledge and data models used to describe the relationships among sensors, algorithms, and missions. Most algorithms are designed for specific sensor systems in anticipation of performing a specific task. Designing and deploying tightly integrated systems limits their potential reuse for new, unanticipated tasks without re-engineering the systems [1–6]. This paper will review the authors’ prior work [7], which addresses the issue of autonomously matching sensor systems to compatible algorithms. Section 2 of the paper will review the challenges of assigning the matched systems to subtasks of missions. Section 3 will review related work of systems and frameworks that assign systems to missions. The remainder of the paper will focus on the authors’ extension of their previous work to now include assignment of the synthesis of systems to subtasks of missions in the context of a persistence surveillance sensing environment. Section 4 discusses the operation of the persistence surveillance environment and Sections 5, 6, and 7 discuss the extended ontological problem-solving framework laboratory prototype for mission assignment and execution.

### Previous Work by Qualls and Russomanno

**Figure 1.**
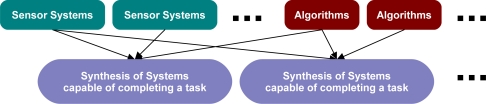
Process for matching sensor systems to compatible algorithms to form a synthesis of systems capable of satisfying a task.

Matching sensor systems to compatible algorithms to form a synthesis of systems poses a significant challenge to problem-solving frameworks. Frameworks must be able to match the systems together and then reuse the same systems in new matches as depicted in Figure 1. In prior work, Qualls and Russomanno [7] focused on the reasoning process of matching sensor systems and algorithms to form a synthesis of systems capable of satisfying a task.

The prior work by the authors included developing a laboratory prototype ontological problem-solving framework that leveraged knowledge engineering techniques to opportunistically infer the discovery and matching of sensor systems to compatible algorithms. The knowledge engineering techniques included an ontology, rules, and inference engine to autonomously form the synthesis of systems. Standard database technologies and SQL queries could have been used for the prototype development, but one of the main shortcomings limiting the matching of systems together is the lack of knowledge models to describe and represent the systems. The knowledge models themselves must leverage well-defined semantics in a machine-interpretable format for autonomous matching. The use of knowledge models also provides the added benefit of more readily transferring the knowledge to other systems as compared to other techniques.

To autonomously form the synthesis of systems, the prototype framework used ontologies to describe properties and relationships among sensor systems, algorithms, and possible synthesis of systems. The ontologies have two parts: (i) the class hierarchy for describing relations among different types of sensor systems; and (ii) algorithms and properties for describing specific properties of each class. Data-type properties, which may be regarded as attributes, are used to describe sensor system and algorithm parameters, such as pixel resolutions, field of view, data format, algorithmic process, and network connections. Object-type properties, which may be regarded as associations, were used to link specific sensor systems and algorithms together during the inference process for the synthesis of systems. With the properties in the ontology, instance data may then be created to represent actual sensor systems and algorithms.

**Figure 2.**
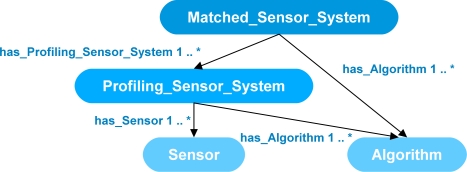
Excerpt of the ontology in the ontological problem-solving framework for matching sensors to algorithms to form a synthesis of systems.

Figure 2 shows a small excerpt of the ontology including four main classes for synthesis: *Matched_Sensor_System*, *Profiling_Sensor_System*, *Sensor*, and *Algorithm*. The *Sensor* class describes a sensing device and the *Algorithm* class describes an algorithmic process. The process can either operate on data generated by the sensing device or data generated by other processes. The *Profiling_Sensor_System* class represents a synthesis of systems that describes possible combinations of *Sensor* and *Algorithm* instances which produce formatted signal profiles of objects as they pass through a sensor system’s field of view. The class *Matched_Sensor_System* describes a synthesized system that contains possible combinations of *Profiling_Sensor_System*, *Algorithm*, and *Sensor* instances, which produce results, such as visualizations or classifications of the generated signal profiles. Not shown is the class hierarchy for the *Target* class, which contains further subclasses of *Human*, *Animal* and *Vehicle.* These subclasses are further refined and include subclasses, such as of *Bird*, *Large_Animal*, and *Bear* for *Animal* and subclasses of *Car*, *Light_Truck*, and *Heavy_Truck,* for *Vehicle*, and so on. Also not shown is a class hierarchy of the *Object_Of_Interest*, which includes subclasses and properties describing accessories, such as backpacks and an extensive description of weapons, which include bladed, non-bladed weapons, and projectile weapons, such as small and heavy arms including pistols, machine guns, and rocket-propelled grenades. Each of the further subclasses has its own respective data-type properties describing those classes. Rules in the form of queries with conditional actions were developed to be processed by an inference engine to search the instance data for possible synthesis of systems. For further information on the development of the ontology in Figure 2, class hierarchy, and rule design please refer to Qualls and Russomanno [7].

**Figure 3.**
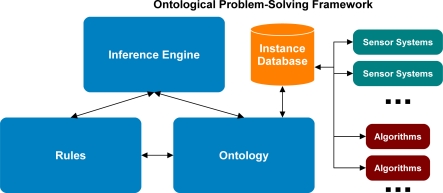
Overview of the laboratory prototype ontological problem-solving framework.

The ontological problem-solving framework with the knowledge engineering techniques discussed above was developed with the TopBraid Composer Maestro software environment by TopQuadrant [8]. TopBraid uses the Web Ontology Language (OWL) [9] for authoring ontologies; rules with logical conditions were developed with SPARQL [10]; and the TopSPIN inference engine was used for processing the SPARQL rules. The authors chose TopBraid Composer Maestro due to their familiarity with this platform from other research projects. OWL is based upon one specific description logic (DL) with the main difference being the naming nomenclature. For example, in OWL a class is a concept in DL, an OWL property is a role in DL, and an OWL object is an individual in DL. The rules developed by the authors in the ontological problem-solving framework are expressed as SPARQL queries with additional constraints on RDF triples. In addition, some of the rules include actions implemented by invoking Java functions via procedural attachment [11]. Other ontological development environments could have been used for the prototype development, such as Protégé with JESS and SWRL [1–7]. Figure 3 shows the overall framework of the ontological problem-solving system.

## 2. Assigning Systems to Missions

**Figure 4.**
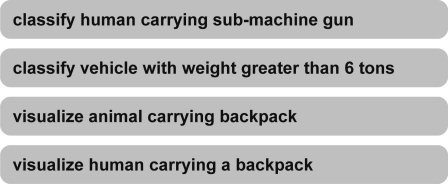
Typical missions for persistence surveillance.

The prior prototype ontological problem-solving framework developed by the authors only matched sensors and algorithms to form a synthesis of systems [7]. The next logical step was to extend the framework to allow for missions to be instantiated on the framework and then autonomously assign the synthesis of systems to the missions. Before an extension could be made, the concept of a mission must be developed. Knowledge acquired from subject matter experts (SMEs) in the fields of sensor system design, algorithm development, and concept of operations (CONOPS) contributed to the development of the concept of missions. The authors elicited knowledge from the SMEs to first develop missions associated with typical persistence surveillance applications as illustrated in Figure 4.

**Figure 5.**
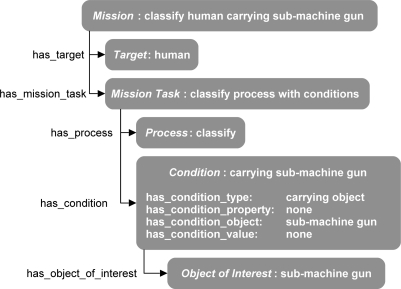
Mission decomposed into a specification via an instance diagram.

The authors and SMEs then analyzed the typical missions to yield a set of specifications that could be used to describe the missions. The specifications included a *target* that must be detected, such as a human or vehicle, and a *mission task* for describing a subtask that describes a *process* and *condition* which takes place on the *target*. A *process* describes what must happen on the detected target, such as “classification”, “visualization”, or “signal profile generation”. The process may have ancillary *conditions*, such as target is carrying a “weapon” or “backpack” or even a *condition* of target has “weight greater than six tons”. A specification is shown in Figure 5 via an instance diagram.

**Figure 6.**
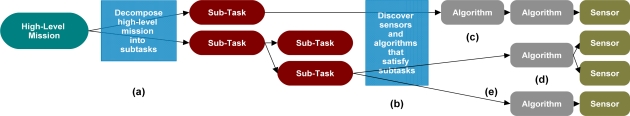
(**a**) Decompose missions to separate subtasks; (**b**) Discover sensors and compatible algorithms, which can complete subtasks; (**c**) Subtask assigned to a chain of algorithms operating on raw data produced by a sensor; (**d**) Subtask assigned to an algorithm operating on raw sensor data from two different sensors; and (**e**) Subtask assigned to an algorithm operating on raw data produced by a sensor.

With the high-level missions decomposed into a set of mission specifications, a problem-solving approach must then assign matched sensor systems and algorithms to the subtasks which satisfy the mission specification as indicated in Figure 6. The problem-solving approach must discover which systems can satisfy the given subtasks as illustrated in Figure 6(b). Once systems have been discovered, the interoperation of multiple sensors and algorithms must be coordinated to perform a subtask as indicated in Figure 6(c–e). To perform these operations, the ontological framework must describe relationships among the sensors, algorithms, and missions in terms of how the subtasks relate to the mission, relationships among subtasks and compatible sensors and algorithms, and the relationships between the sensors and algorithms. Section 5 details how the developed concept of a mission was integrated into the authors’ previous work.

## 3. Related Work

Various approaches have been designed and engineered to assign sensors and algorithms to missions or task specifications, such as Semantic sensor mission assignment [12], Ontological sensor mission assignment [13], Knowledge base for sensors to missions [14], GloServ [15], Ontology Centric sensor mission assignment [16], Resource management [17], Sensor Mission Matching [18], Semantic-aware cooperative agents [19], Query Processing for sensor networks [20], Agilla [21–23], Geographic Information System Framework [24,25], Semantic Streams [26,27], and Sensor OASiS [28]. Relevant to our work is the development of ontologies that represent and describe sensor systems such OntoSensor [2–6], Sensor Network Data Ontology [29], Sensor and Data Wrapping Ontology [30], Stimulus-Sensor-Observation Ontology [31], Sensor Observation and Measurement Ontology [32], Semantic Sensor Network Ontology [33], and Disaster Management Sensor Ontology [34]. Also of importance to the authors’ work are other examples of relevant sensor ontologies [35]. There are many logical models and standards to follow and adapt, such as the Sensor Modeling Language (SensorML) [36], that leverage the Unified Modeling Language (UML) to conceptualize sensor systems and algorithms to facilitate interoperability. Also, the Open Geospatial Consortium (OGC) [37] drafts standards that may be used to define metadata encodings and interoperability interface standards to facilitate problem-solving frameworks that can integrate sensor systems and algorithms into information infrastructures. The OGC includes many standards, such as Observations and Measurements (O&M) [38,39], SensorML [40], Transducer Model Language (TML) [41], Sensor Observation Service (SOS) [42], Sensor Planning Service (SPS) [43], Sensor Alert Service (SAS) [44], and Web Notification Services (WNS) [45].

**Figure 7.**
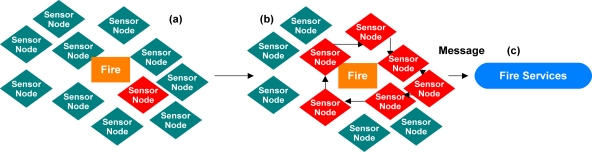
(**a**) Fire detection protocol on a node in an Agilla sensor network that detects a fire; (**b**) Protocol activates other protocols on different nodes to determine the perimeter of the fire and then activates other protocols for fire services; and (**c**) Activation of fire services.

**Figure 8.**
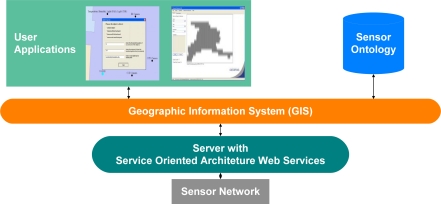
Geographical Information System Framework displaying interconnections among the geographic information system, service-oriented architecture, ontologies and end user software applications.

One example system, Agilla [21–23], is a framework used to monitor sensor systems connected to a sensor network. Agilla uses protocols with specific conditions that, when met, will perform a specific action or actions. For example, the actions and conditions may be to activate other protocols when a sensor reports a temperature above a specific threshold. The newly activated protocols may then coordinate other sensors to collect data, invoke algorithms for further analysis, or even activate more protocols to perform a specific action or actions. Figure 7(a) shows an Agilla network with a fire detection protocol on one sensor node. The fire detection protocol has the task of detecting a temperature above a specific threshold. Once the temperature threshold is reached, the protocol will activate other fire detection protocols on more sensors nodes, Figure 7(b). As the protocols activate on the other sensor nodes, the protocols will determine the perimeter of the fire and then send the perimeter data to a new protocol, Figure 7(c), which then activates fire services [21–23]. Another example system, Geographical Information System Framework [24,25], leverages several different frameworks in the overall management of sensor systems and algorithms on a sensor network as depicted in Figure 8. The framework includes knowledge models, such as ontologies, for describing sensors, algorithms, and tasks. Service-oriented architectures are used to handle communications among all systems, and geographic information system placement logic is used for tasking. The different frameworks operating together allow end users or autonomous systems to query the framework for available sensor systems and then task the sensor systems to retrieve data or to perform specific actions based on sensor data [24,25].

## 4. Persistence Surveillance Sensing Environment

To demonstrate the efficacy of the extended ontological problem-solving framework, a persistence surveillance sensing environment was constructed from a family of emulated unattended ground profiling sensor systems and algorithms. The profiling sensors provide a means for capturing signals of objects which pass through a profiling sensor’s field of view. The signals are then passed to algorithms which create profiles of the signals, which are then sent to other algorithms for further processing, such as object classification or visualization. The profiling sensors have a common theme in that they are low cost and provide a signal that can be classified. The profiling sensors are denoted by the nomenclature PFx [46]. The PFx sensors may use a variety of sensing bands, including visible, near infrared, short-wave infrared, mid-wave infrared, and long-wave infrared bands. They typically share a common design principle of using a sparse detector array.

**Figure 9.**
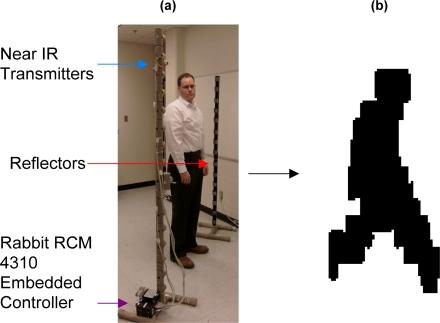
(**a**) PFx sensor system using sixteen near-infrared detectors deployed vertically with no horizontal displacement; and (**b**) Silhouette generated by an algorithm operating on the sensor data.

Figure 9(a) shows a sparse detector PFx sensor consisting of sixteen near-infrared detectors in a vertical deployment with no relative horizontal displacement and a reflector pole. When an object passes between the two poles, which is the field of view of the sensors, the resulting signal will be recorded. An algorithm then processes the signal by formatting the raw sensor data using run-length encoding. The formatted sensor data may be used by other algorithms to visualize the acquired data as a silhouette shown in Figure 9(b). Other possible configurations of a vertical near infrared sparse detector may include a horizontal displacement, which may be used to determine the velocity of an object [46–53]. The chain of creating raw sensor data, generating profiles, and then processing the profiles for visualization or classification provides a unique opportunity to show how the prototype ontological framework can autonomously assign the PFx sensors and algorithms to the subtasks of various missions based on their relationships and capabilities.

## 5. Reasoning Process for Assigning Sensor Systems and Algorithms to Missions

### 5.1. Problem-Solving Framework for Assigning a Synthesis of Systems to Mission Specifications

**Figure 10.**
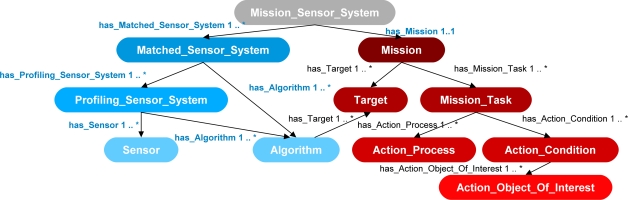
Extension of the prototype ontological problem-solving framework for matching sensor systems to algorithms to form a synthesis of systems that may now be assigned to subtasks of missions.

To address the challenge of assigning sensor systems and algorithms to high-level missions the previous work by Qualls and Russomanno [7] was extended with the concept of a mission developed from eliciting knowledge from SMEs. Figure 10 shows the original ontology of the problem solving-framework, as seen in Figure 3, extended with an ontology for describing mission specifications. The extended ontology is shown here with two additional classes: *Mission_Sensor_System* in gray, and *Mission* in red. The *Mission* class has five supporting classes, also in red, to describe mission specifications: *Target*, *Mission_Task*, *Action_Process*, *Action_Condition*, and *Action_Object_Of_Interest*. The primary goal of the ontology in the prototype ontological framework is to support the synthesis of the *Mission_Sensor_System*, which is a synthesis of systems assigned to a mission.

A *Mission_Sensor_System* class describes a possible combination of *Matched_Sensor_System* instances and a *Mission* instance through the two object type properties has_Matched_Sensor_System and has_Mission. A *Mission_Sensor_System* may have many *Matched_Sensor_System* instances but one only *Mission* instance. The *Mission* class describes the various specifications of a mission. The *Mission* class leverages two other classes, *Target* and *Mission_Task*, to define mission specifications. The *Target* class describes the object that the mission needs to detect, such as human or animal. The *Mission_Task* class describes the process and condition which must take place on the *Target* instance. To define the process specification, the *Mission_Task* leverages two other classes: *Action_Process* and *Action_Condition*. The *Action_Process* class describes a specification process, such as “classify” or “visualize” for the detected *Target* instance. The *Action_Condition* class describes further specifications that the *Action_Process* might require. Last, the class *Action_Object_Of_Interest* describes objects that a *Target* instance may be associated with, for example, objects that may be carried by a human or animal.

### 5.2. Ontological Framework Rules

The original prototype ontological problem-solving framework used SPARQL [10], a graph-matching query language to implement the rules to query the instance data and return possible synthesis of systems. SPARQL rules can be regarded as Horn clauses with addition logical constraints. The rules contain the following two components; CONSTRUCT and WHERE clauses. First, the CONSTRUCT clause returns possible object instances, which contain new properties, derived properties, and links to other class instances and their corresponding attributes. Second, the WHERE clause contains statements that specify constraints. The constraints include the properties that must exist and the logical constraints that properties of a class instance must satisfy before the rule will execute. Each of the constraints in a single rule are connected via a logical conjunction (logical AND), whereas a collection of rules of a common theme are connected via a logical disjunction (logical OR). Once all properties and logical constraints of the WHERE clause are satisfied, the corresponding CONSTRUCT clause will return the possible object instance or instances.

**Figure 11.**
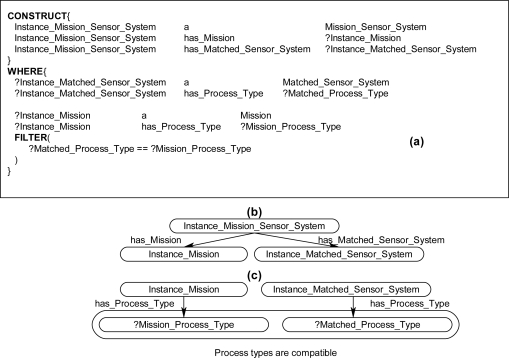
(**a**) Example SPARQL rule with CONSTRUCT and WHERE clauses, which returns a possible *Mission_Sensor_System* instance; (**b**) Instance diagram of CONSTRUCT clause; and (**c**) Instance diagram of WHERE clause.

New rules were developed for assigning the synthesis of systems to missions, thus returning possible *Mission_Sensor_System* instances. Figure 11 shows an example SPARQL rule that queries the existing instance data and returns a *Mission_Sensor_System* instance in the CONSTRUCT clause when the properties and logical constraints are satisfied in the WHERE clause. The CONSTRUCT clause in Figure 11(a) contains three statements. The first statement declares the object instance Instance_Mission_Sensor_System to be of class type *Mission_Sensor_System*. The second two statements establish links to the possible existing instances through the two properties: has_Mission and has_Matched_Sensor_System. To establish these two links, the two properties are linked to two variables Instance_Mission and Instance_Matched_Sensor_System, respectively. The WHERE clause in Figure 11(b) contains five statements. In the first two statements, the object variable Instance_Matched_Sensor_System is instantiated with an instance of class type *Matched_Sensor_System* and the variable Matched_Process_Type is instantiated with the value from the data type property has_Process_Type from the same *Matched_Sensor_System* instance. In the second two statements, the object variable Instance_Mission is instantiated with an instance of class type *Mission* and the variable Mission_Process_Type is instantiated with the value from the data type property has_Process_Type from the same *Mission* instance. The final statement in the WHERE clause contains the FILTER command which appears as a simple logical constraint that compares two variables. This particular FILTER command compares the two data type variables Matched_Process_Type and Mission_Process_Type for equality. When the inference engine processes this rule, the CONSTRUCT clause will return a possible *Mission_Sensor_System* instance with links to a *Matched_Sensor_System* instance and links to an assigned *Mission* instance if the two instances and properties exist and if the two properties are equal in the WHERE clause. Once a *Mission_Sensor_System* instance has been returned by the rules, the ontological framework can then execute the mission by coordinating all synthesized systems, sensors, and algorithms assigned to that mission and returning the results of the mission via a procedural attachment statement.

One note of interest is the subsumption qualities of the logical constraint for the FILTER command. For example, if the Matched_Process_Type variable is set to a subclass of the Process_Type and the Mission_Process_Type variable is set to a superclass of the Process_Type, the rule will need to return true or false depending on a set threshold for the semantic distance between the variables. To set this threshold the authors decided to set the mission as a priority. The reason for this selection is based on feedback from the SMEs in terms of CONOPs. For example, a mission may be created for a specific target but no synthesis of systems can complete that exact mission. Forcing the framework to assign systems only to exact matches of missions would severely limit the capabilities of the framework. So the authors decided to allow the framework to return possible best matches between a synthesis and a mission. For the ontological framework the semantic distance threshold has been set as follows for the prototype. There are two basic conditions: if the property of the mission is a subclass of the matched system or if the property of the mission is a superclass of the match system. First, if a mission property such as has_Action_Object_Of_Interest is set to a value that is a subclass of the same property of the matched systems, the framework will assign the matched system to the mission up to the top-level superclass that property may have. For example, if the mission has_Action_Object_Of_Interest variable is set to pistol, the framework would assign matched systems up to highest class domain of the property in this case has_Action_Object_Of_Interest, which is the class Object_Of_Interest. Second, if the has_Action_Object_Of_Interest variable of the matched system is set to a value that is a subclass of the same property of the mission then an assignment will take place. For example, if the mission has_Action_Object_Of_Interest property is set to pistol, the framework would assign matched systems that are subclasses of pistol.

**Figure 12.**
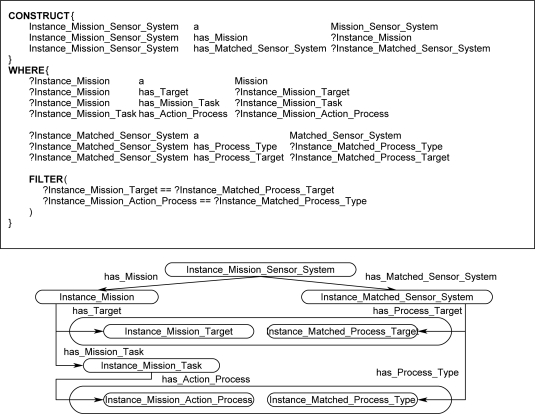
Rule and instance diagram showing how a *Mission_Sensor_System* is returned for a simple *Mission* if a *Matched_Sensor_System* can accomplish the mission based on *Action_Process* and *Target* properties.

**Figure 13.**
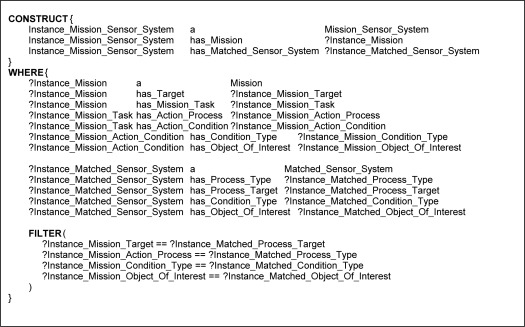

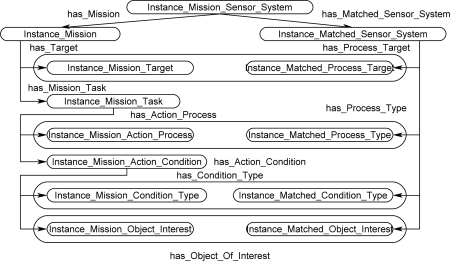
Rule and instance diagram showing how a *Mission_Sensor_System* is returned for an advanced *Mission* if a *Matched_Sensor_System* can accomplish the mission based on *Action_Process*, *Action_Condition*, and *Target* properties.

The rules in the ontological problem-solving framework all follow a similar structure outlined in Figure 11. The rules bind on all combinations of *Mission* and *Matched_Sensor_System* instances and return possible *Mission_Sensor_System* instances in the CONSTRUCT clause when the corresponding properties exist and logical constraint statements are met in the WHERE clause. Figures 12 and 13 each show one of many different kinds of rules that return possible *Mission_Sensor_System* instances and their resulting instance diagrams. These rules bind on properties of the *Matched_Sensor_System* instance that link back to other instances, such as the type of process the system can accomplish, and additional properties, such as conditions on the process that may or may not be optional. The rules also bind on properties of a *Mission* instance, which, as previously discussed, include *Target*, *Mission_Task*, *Action_Process*, and *Action_Condition*. Figure 12 shows a rule which binds on a simple mission to process a target with no conditions, such as “classify human male”. Figure 13 shows a rule that binds on more advanced missions that processes a target with conditions, such as “visualize horse carrying backpack” or “classify human male with height greater than six feet”, respectively.

## 6. Example of Assigning a Synthesis of Systems to Mission Specifications

**Figure 14.**
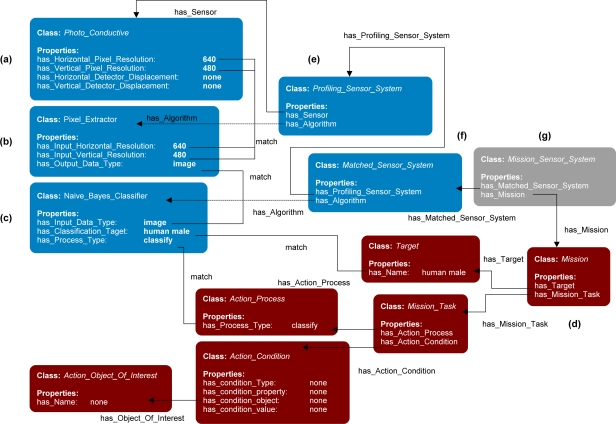
*Mission_Sensor_System* instance diagram showing a linked *Mission* instance “classify human male” matched to a synthesized system capable of satisfying the high-level mission. (**a**) *Sensor* instance *Photo_Conductive*; (**b**) *Algorithm* instance *Pixel_Extractor*; (**c**) *Algorithm* instance *Naive_Bayes_Classifier;* (**d**) *Mission* instance “classify human male”; (**e**) *Profiling_Sensor_System* instance; (**f**) *Matched_Sensor_System* instance; and (**g**) *Mission_Sensor_System* instance.

To show how the prototype ontological problem-solving framework operates, a small example has been created. Figure 14 shows an overview of all emulated assets instantiated and the resulting synthesis of systems and assignment to a mission instance. To begin, the ontological framework was instantiated with: (i) one emulated sensor systems *Photo_Conductive* (Figure 14(a)); (ii) two algorithms, *Pixel_Extractor* (Figure 14(b)) and *Naive_Bayes_Classifier* (Figure 14(c)); and (iii) and one mission instance (Figure 14(d)). The following section will detail how the sensor system and algorithms are matched together to form a synthesis of systems that are assigned to a mission. Each of the instances has many different data-type properties, but for this example only a few relevant properties are show in Figure 14.

The *Sensor* instance *Photo_Conductive* has four properties; has_Horizontal_Pixel_Resolution set to 640 pixels, has_Vertical_Pixel_Resolution set to 480 pixels, has_Horizontal_Detector_Displacement, and has_Vertical_Detector_Displacement both set to none. The *Photo_Conductive* instance represents a sensor capable of generating a signal profile of a passing target. The *Algorithm* instance *Pixel_Extractor* has three properties; has_Input_Horizontal_Resolution set to 640 pixels, has_Input_Vertical_Resolution set to 480 pixels, and has_Output_Data_Type set to image. The *Pixel_Extractor* instance represents an algorithm capable of loading a raw signal profile data in 640 × 480 format and then generating a formatted signal profile into an image format. The second *Algorithm* instance *Naive_Bayes_Classifier* has three properties: (i) has_Input_Data_Type set to image; (ii) has_Classification_Target set to human male; and (iii) and has_Process_Type set to classify. The *Naive_Bayes_Classifier* instance describes a classifier that operates on features of an image and then classifies the image as a human male or not a human male.

The Mission instance represents a mission that requires the detection of human males, *i.e.*, classify human male. The *Mission* instance has two object-type properties, has_Target and has_Mission_Task, which link to the *Target* instance and *Mission_Task* instance. The *Target* instance describes a human male instance that has many properties, such as has_Name and not shown has_Height, and has_Weight. The instance *Mission_Task* has two object-type properties has_Action_Process which links to the instance *Action_Process* “classify” and the property has_Action_Condition which links to the *Action_Condition* instance “none”. The *Action_Process* instance has many data-type properties, such as has_Process_Type, which can have the values classify, profile_generator, convertor, and visualizer. For this case, the data-type property is set to classify. The *Action_Condition* instance “none” has three data-type properties, has_Condition_Type, has_Condition_Property, and has_Condition_Value, each set to “none” and one object-type property, has_Condition_Object, which links to the instance *Action_Object_Of_Interest* “none”. The instance Action_Object_Of_Interest “none” is of type *Object_Of_Interest* which describes a possible object the *Target* may be holding or wearing, but in this example, the mission does not specify if the human male is carrying an object, so all values are set to none.

With all systems and a mission instantiated on the prototype ontological framework, rules such as those in Figures 12 and 13 will process the instance data to form a synthesis of systems and assign the synthesis to the mission. The first synthesis of systems to be returned is a *Profiling_Sensor_System* instance shown in Figure 14(e). The *Profiling_Sensor_System* instance was returned because the properties of *Photo_Conductive* and *Pixel_Extractor* matched, *i.e.*, the output pixel resolutions of the *Photo_Conductive* and input pixel resolutions of the *Pixel_Extractor* matched to 640 × 480. The synthesized *Profiling_Sensor_System* instance contains two derived object-type properties that link to the *Sensor* instance Ph*oto_Conductive* and the *Algorithm* instance *Pixel_Extractor*, called has_Sensor and has_Algorithm. The *Profiling_Sensor_System* instance represents a synthesis of systems capable of formatting a raw signal profile into a formatted “image” profile.

The next pass of the inference cycle will produce the second synthesis of systems; the *Matched_Sensor_System* instance shown in Figure 14(f). The *Matched_Sensor_System* instance contains the two object-type properties has_Profiling_Sensor_System, which links to the synthesized *Profiling_Sensor_System* instance, and has_Algorithm, which links to the *Naive_Bayes_Classifier* instance. The algorithm *Naive_Bayes_Classifier* was matched to the Profiling_Sensor_System instance because the data-type property has_Output_Data_Type set to “image” matched the data-type property has_Input_Data_Type set to “image”, respectively. The new *Matched_Sensor_System* instance represents a synthesized system, which generates raw signal data that can then be classified as a human male or not a human male.

On the next inference cycle, the rules return a possible *Mission_Sensor_System* instance, shown in Figure 14(g), which assigns the synthesized system *Matched_Sensor_System* instance to the simple *Mission* instance because of two sets of properties. First, the data-type property has_Classification_Target value “human male”, which is linked to the *Matched_Sensor_System* through the has_Algorithm object-type property, matches to the data type property has_Name “human male” in the *Target* instance, which is linked to the instance *Mission* through the object-type property has_Target. Second, the *Naive_Bayes_Classifier* instance has the data-type property has_Process_Type set to the value “classify”. The *Naive_Bayes_Classifier* instance is linked to the *Matched_Sensor_ System* instance through the object-type property has_Algorithm because the data-type property has_Process_Type of the instance *Action_Process* is set to “classify”. *Action_Process* is linked to the instance *Mission_Task* through the object-type property has_Action_Process, which in turn is linked to the *Mission* instance through the object-type property has_Mission_Task. The synthesized *Mission_Sensor_System* instance links to the synthesized *Matched_Sensor_System* instance through the object-type property has_Matched_Sensor_System and links to the *Mission* instance through the object-type property has_Mission and represent synthesized systems ready to be coordinated to complete the mission classify human male. The returned *Mission_Sensor_System* system is added as an instance in the ontology so further inference can leverage the synthesis of systems and mission for further complex mission tasking or for actual coordination to execute the mission. Although Figure 14 shows relatively simple properties, and the rules in Figure 12 and Figure 13 bind on simple compatibility constraints, further properties and more complex uses of the SPARQL, FILTER, and OPTIONAL commands may allow for more complex synthesized systems to be returned and assigned to increasingly sophisticated missions.

## 7. Instantiated Emulated Profiling Sensor Systems and Algorithms

**Figure 15.**
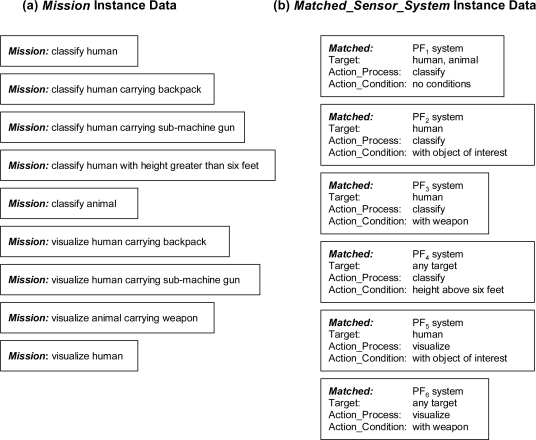
Instantiated examples on the ontological framework: (**a**) Nine different *Mission* instances consisting of detection and classification of targets and visualization of targets; and (**b**) Six different *Matched_Sensor_System* instances with links to *Profiling_Sensor_System*, *Sensor* and *Algorithm* instances matched together to form a synthesized system capable of performing a task.

To show the efficacy of the ontological problem-solving framework, several emulated profiling sensor systems and algorithms were instantiated as complete *Matched_Sensor_System* instances in the ontological problem-solving framework as a prototype environment for testing. In the prototype environment, nine different *Mission* instances and six different *Matched_Sensor_System* instances were instantiated, Figure 15. Each of the various *Matched_Sensor_System* instances contained *Profiling_Sensor_System* instances made up of matched emulated sensor systems and algorithms, with some of the emulated systems shared between different *Matched_Sensor_System* instances.

**Figure 16.**
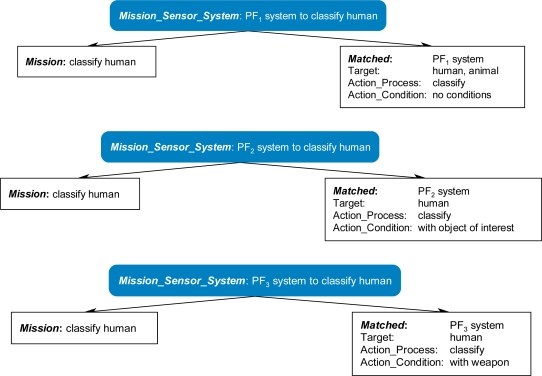

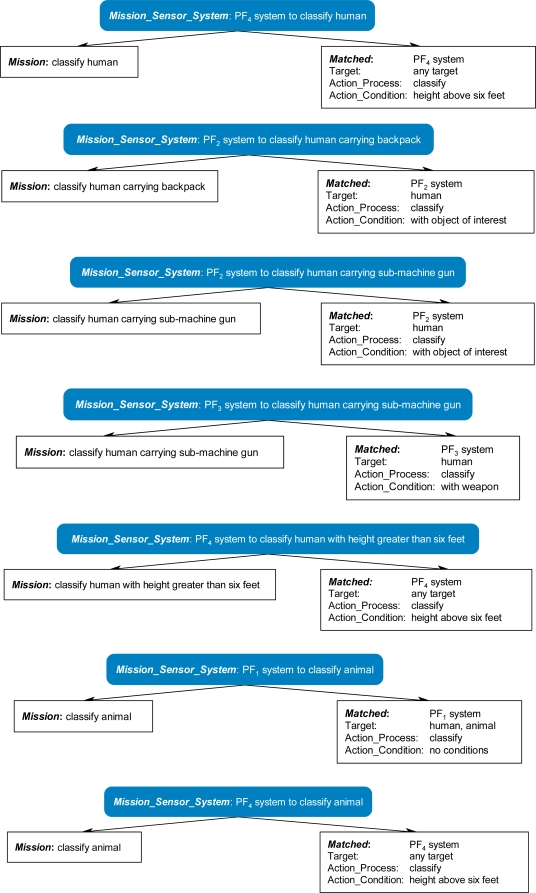

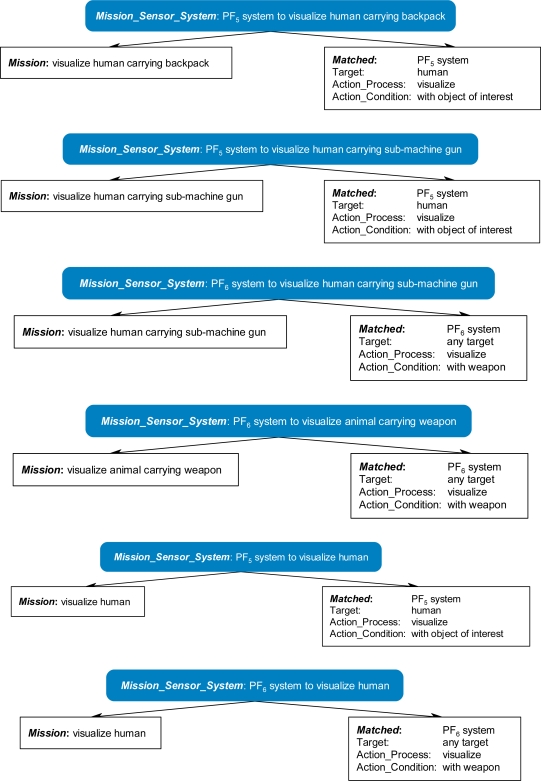
Sixteen new *Mission_Sensor_System* instances were returned with derived relationships after the inference cycle completed.

When the ontological problem-solving framework begins, the inference cycle processes the rules similar to those in Figures 12 and 13. When the inference cycles terminate, sixteen new *Mission_Sensor_System* instances were returned as shown in Figure 16. From Figure 16, multiple *Matched_Sensor_System* instances were matched to a single *Mission* instance while in some cases a single *Matched_Sensor_System* instance was matched to multiple *Mission* instances. For example, the *Mission* instance “classify human carrying sub-machine gun” can be completed by two different *Matched_Sensor_System* instances: “PF_2_ system” and “PF_3_ system” because the Action_Process “classify” of the *Mission* matched the Process_Type “classify” of both PFx systems and each of the *Mission* instance Action_Object_Of_Interest “sub-machine gun” matched the Action_Condition of both PFx systems. This particular assignment also represents how the authors chose to handle subsumption with a semantic distance threshold in that the Action_Condition of both PFx systems were not “sub-machine gun” but “object of interest” and “weapon”, which are each super classes of “sub-machine gun”. Assigning matched systems in this way allows the ontological framework to “best fit” a mission to a synthesis of systems. Also the *Matched_Sensor_System* instance “PF_5_ system” can complete three different *Mission* instances: “visualize human carrying backpack”, “visualize human carrying sub-machine gun”, and “visualize human”.

Once the assignments have been completed, *i.e.*, the *Mission_Sensor_System* instances have been returned by the inference engine, the prototype ontological framework selects a single completed *Mission_Sensor_System* instance through a rule and then coordinates all sensor systems and algorithms associated with the instance via a procedural attachment within the rule to complete the mission and return the results. Once the mission has been completed, the ontological framework will then select the next *Mission_Sensor_System* instance to coordinate, complete, and return the results. Since the ontological problem-solving framework is in a laboratory prototype stage, only a single mission is completed at a time. Also, if the *Mission* instance is matched to several *Matched_Sensor_System* instances, the same mission will be completed for each assignment regardless if it was previously completed. Improved systems can be developed that allow for simultaneous mission coordination, completion, and avoidance of repeating a mission, but the focus here is on proof-of-concept.

## 8. Discussion

The challenge for the ontological problem-solving framework was to assign a synthesis of systems to subtasks of mission specifications. Even though the rules described in this paper contain relatively simple compatibility constraints among sensors, algorithms, and missions, these rules illustrate an important proof-of-concept. Namely, a problem-solving approach to matching *Sensor* and *Algorithm* instances to form synthesized *Matched_Sensor_System* and *Profiling_Sensor_System* instances which are then assigned to high-level Mission instances to form a *Mission_Sensor_System* instance that is ready to be executed by the ontological framework or other autonomous systems for mission completion. It is important to note that multiple *Matched_Sensor_System* instances were reused and assigned to different *Mission* instances, which the *Matched_Sensor_System* instances were capable of satisfying. For example, the *Matched_Sensor_System* PF5 instance, which is capable of visualizing a human carrying an object, was assigned to three different *Mission* instances that required the detection of “humans” with and without various objects, such as “weapons” or “backpacks”. It is important to realize that the *Mission_Sensor_System* is more than just sensors and algorithms assigned to a mission, the *Mission_Sensor_System* is a synthesized system, which is capable of performing the assigned subtasks to satisfy the overall mission specification and returning results for further analysis or more complex missions.

Rules in the ontological framework may operate on more than just properties of the various instances. For example, more complex rules may determine that certain *Mission_Sensor_System* instances may be composited together to satisfy more complex mission specifications. Possible complex *Mission* instances may include the detection of multiple targets and the tasking of other complex synthesized systems to monitor the targets for a specific time, which could be represented as a single *Mission_Sensor_System* instance. Other rules may even generate new missions or decompose missions into specifications for subtask assignment. Without leveraging ontologies, rules, the inference engine, and the concept of synthesized systems, all of the sensors and algorithms would need to be configured *a priori* for the anticipated missions and reconfigured for unanticipated missions. Most missions and subtasks are not known at the time of system deployment, therefore, a problem-solving approach may opportunistically assign synthesized systems to subtasks of high-level missions in real-time, which is an extremely important capability for dynamically changing requirements in a particular environment.

As discussed in the related work, many framework and middleware systems have been researched and developed to assign systems to missions. Some of the middleware systems used *a priori* matching of assets to mission tasks, such as Agilla [21–23], which limits the reuse of assets for other tasks without new matching occurring *a priori*. Other systems that use knowledge bases for sensor mission assignment [12–19] leveraged ontologies and other techniques for automated sensor mission assignment. As stated previously, the reasoning and use of knowledge engineering techniques by the authors for the prototype ontological framework is similar to other efforts in some aspects. The work described in this paper differs from these other works in that the domain of the missions and assets were limited to a persistence surveillance sensing environment. By limiting the domain, the research of the prototype ontological problem-solving framework could focus on providing a complete solution that not only assigns assets to missions but also includes a coordination system that connects to emulated assets and completes the mission.

Although the priority at this stage of this research is the logical problem-solving framework, another important aspect is performance. Performance can be analyzed along several dimensions, including scale-up analysis with solution finding, mission operation time, and mission completion rates. First, scale-up performance analysis is limited at this point, but the ontological framework can scale to a very large number of instantiated sensors, algorithms and missions, limited only by physical memory constraints. The reasoning strategy used by the inference engine, along with the features expressible in the knowledge representation language, dictate the overall computational complexity, which in turn determines the time for the ontological framework to infer all combinations of sensors, algorithms, and missions. Performance can be increased by enabling the inference engine to check multiple sensors, algorithms, and missions in parallel or by invoking the inference engine multiple times in parallel, while having a strategy in place to eliminate redundant bindings.

Currently, the prototype ontological framework has been tested with fifty systems not detailed in this paper. The ontological framework takes less than two seconds to find all possible combinations, which equates to over 500 new combinations after the inference cycle completes. As more sensor systems and algorithms are added to the ontological framework, combinatorial explosion becomes an issue. Combinatorial explosion may be somewhat mitigated once asset resource control and time constraints are taken into account in the rules. First, as the number of systems increase, new systems for the ontological framework will need to be researched and developed that limit the number of solutions found and to determine the correctness of the proposed solution. Second, mission operation time is determined by how the ontological framework or other systems can execute and complete missions. To increase performance, the framework needs to operate missions in parallel and prioritize matched sensors and algorithms, which are assigned to different missions. Third, the prototype ontological framework described in this paper does not take into account competing missions, *i.e.*, resource management. New research is focusing on designing systems that provide information to the ontological framework for priority of missions, such as time constraints, availability of sensor systems and algorithms, *i.e.*, resource control and time difference between collection of data and mission time completion. Other mechanisms will need to be established that prevent a mission from never completing. For example, a mission may be to classify humans, but humans may never be detected thus locking the resources for that mission indefinitely. Placing time constraints on active missions may prevent the never ending mission.

## 9. Conclusions

Although the paper only shows PFx sensors, algorithms, and missions related to operation of those sensors and algorithms instantiated on the ontological framework, the principles and techniques that have been demonstrated may be appropriate for other types of sensors, algorithms, or missions. Development and deployment of new sensor systems and algorithms will continue to create challenges, such as discovering appropriate sensor systems and algorithms to satisfy tasks which may then be assigned to subtasks of mission specifications. The lack of explicit knowledge models used to describe the capabilities of sensor systems and algorithms and the specifications on high-level missions compounds the challenge even further. To allow the flexibility of assigning systems to unanticipated missions, the framework must leverage knowledge models, such as ontologies, rules, and inference engines, in a machine-interpretable format to perform automated synthesis and assignment of sensor systems and algorithms. The use of ontologies facilitates inference with rules allowing the prototype ontological problem-solving framework to autonomously reason about how a synthesis of systems may be formed and then assigned to missions. New research for the prototype is focusing on addressing the issues raised in the discussion section, such as combinatorial explosion, resource constraints, mission completion time, and other areas. The problem-solving approach developed in this paper for the laboratory prototype ontological framework is the first step towards achieving reuse of systems without an *a priori* configuration, flexible assignment of synthesized systems to mission subtasks through automated inference, and addressing further issues affecting a frameworks ability to autonomously coordinate assets.
